# Fallopian canal arachnoid cyst with acute facial nerve paralysis in children: a report of two cases and literature review

**DOI:** 10.3389/fneur.2023.1226404

**Published:** 2023-09-04

**Authors:** Jianbin Sun, Weiju Han

**Affiliations:** ^1^Medical School of Chinese PLA, Beijing, China; ^2^Department of Otorhinolaryngology Head and Neck Surgery, The Six Medical Center, Chinese PLA General Hospital, Beijing, China; ^3^National Clinical Research Center for Otorhinolaryngologic Diseases, Beijing, China

**Keywords:** arachnoid cyst, fallopian canal, facial nerve paralysis, cerebrospinal fluid otorrhea, meningitis

## Abstract

**Introduction:**

Symptoms induced by arachnoid cysts in the fallopian canal are uncommon, and facial nerve paralysis without cerebrospinal fluid otorrhea is comparatively rarer.

**Methods:**

Herein, we present two cases of arachnoid cysts in the fallopian canal with acute severe facial nerve paralysis and review the relevant literature.

**Results:**

The symptoms and imaging findings of these two cases resembled those of facial nerve schwannomas. Cerebrospinal fluid otorrhea occurred upon removal of the arachnoid cyst, and the facial nerve was observed to be separated into multiple filaments or compressed and atrophied. Facial-hypoglossal nerve anastomosis and decompression were conducted after packing the dehiscence of cerebrospinal fluid otorrhea for the two cases.

**Conclusion:**

Arachnoid cysts of the fallopian canal rarely cause facial nerve paralysis. Enhanced magnetic resonance imaging is vital for differentiating schwannomas. Different treatment strategies should be adopted for patients with different degrees of facial nerve paralysis; however, concurrent repair of cerebrospinal fluid otorrhea and facial nerves during surgery can occasionally be challenging.

## 1. Introduction

The facial nerve is surrounded by a dense, collagenous sheath throughout its course in the temporal bone; however, the dual lining of the internal auditory meatus blends with the perineurium of the facial nerve near the fundus of the internal auditory meatus, where a fibrous ring is formed, which permits extension of the subarachnoid space (1). During early facial nerve development, factors such as elevated subarachnoid pressure may extend the cerebrospinal fluid (CSF) space distal to the internal auditory meatus and into the fallopian canal. Persistent elevated subarachnoid pressure may lead to the erosion of the fallopian canal and spontaneous cerebrospinal fluid otorrhea or damage of the facial nerve and even facial nerve paralysis. Spontaneous CSF otorrhea through this path is not common, and only fewer than 20 cases have been reported worldwide over the past several decades (2), with facial nerve paralysis even rarer. Thus far, only three patients with fallopian canal arachnoid cysts have been globally reported to present facial nerve paralysis; nonetheless, the paralysis was either mild or transient, and their principal problems were not facial nerve paralysis but recurrent CSF otorrhea, idiopathic intracranial hypertension, or meningitis (3–5).

Herein, we present two cases of arachnoid cysts in the fallopian canal whose cardinal symptoms were acute severe facial nerve paralysis, which confounded the preoperative diagnosis by mimicking facial nerve schwannoma, and intraoperative CSF otorrhea, which complicated the facial nerve repair.

## 2. Case presentations

### 2.1. Case one

**Figure 1:**
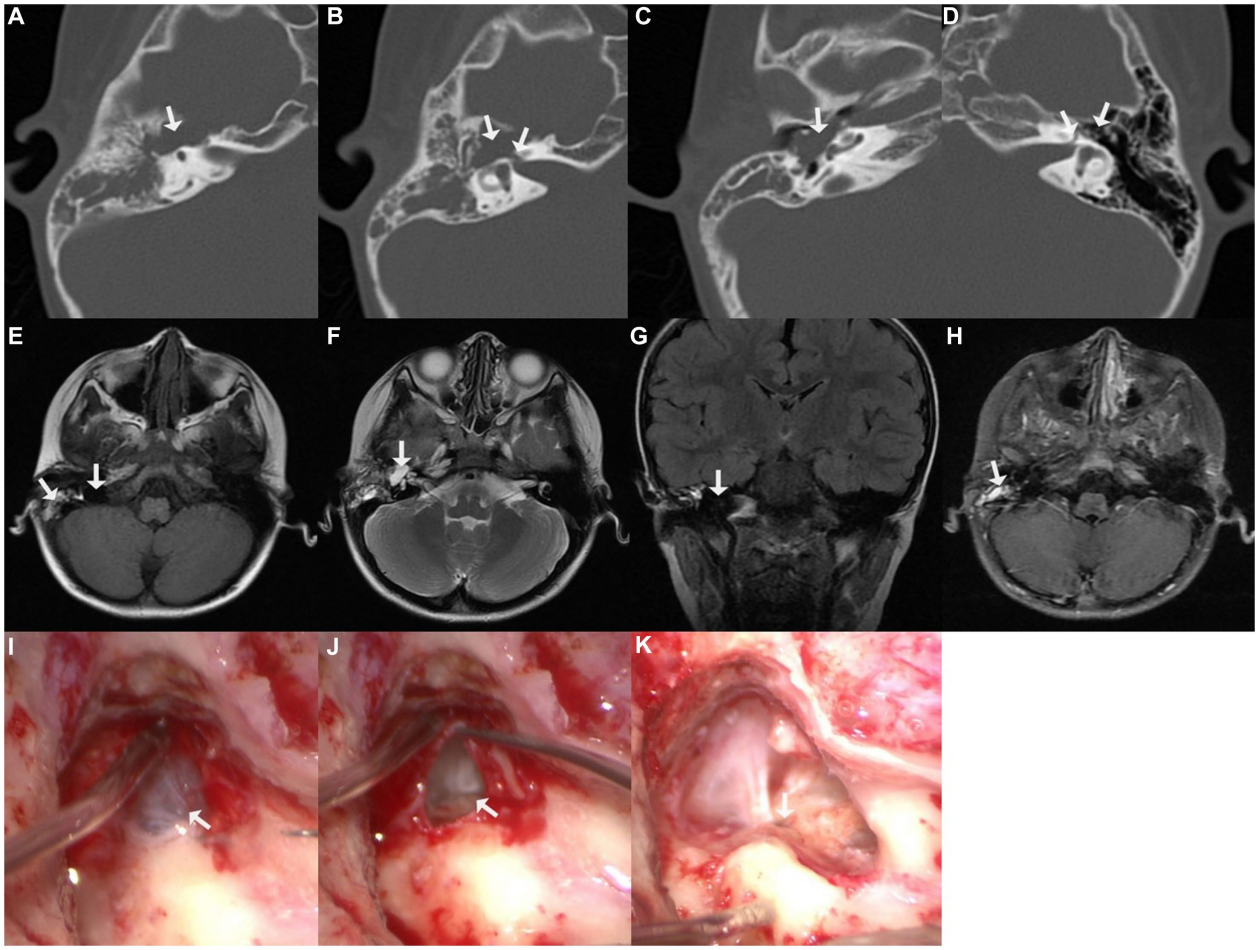
Imaging of case one. **(A–C)** Cystic lesion in the tympanic cavity on axial computed tomographic (CT) imaging; **(B)** enlarged fallopian canal at labyrinthine segment; **(D)** normal fallopian canal and tympanic cavity on the contralateral left side; **(E)** hypointensity of the lesion on T1-weighted imaging (T1WI), with mixed signals of exudate in the mastoid cavity; **(F)** hyperintensity of the perigeniculate lesion on T2-weighted imaging (T2WI); **(G)** hypointensity of the lesion on coronal T2 fluid-attenuated inversion recovery imaging (T2 FLAIR); **(H)** part of the lesion was enhanced on T1-weighted contrast-enhanced imaging; **(I)** exploration revealed a cyst-like lesion was located in the tympanic cavity, along the length of the facial nerve; **(J)** massive cerebrospinal fluid (CSF) leaked was observed upon piercing the cyst; **(K)** CSF was released from the defective fundus of the internal auditory meatus.

A 5-year-old boy visited our center with right-sided complete facial nerve paralysis following a bout of fever and headache 1.5 years prior. He was diagnosed with pneumococcal meningitis and treated with ceftriaxone combined with dexamethasone for approximately 2 weeks at the local children’s hospital. He recovered from meningitis but facial nerve paralysis did not improve; thus, facial exercise and acupuncture were later used for facial rehabilitation, but these did not work out. He finally came to our center for complete right-sided facial nerve paralysis. Physical examination revealed House–Brackmann (HB) grade VI facial nerve paralysis, and electromyography indicated fibrillation potential. Pure-tone audiometry revealed right-sided conductive hearing loss. Computed tomography (CT) indicated an asymmetrically enlarged fallopian canal in the labyrinthine segment of the facial nerve and a cystic lesion in the tympanic cavity. Magnetic resonance imaging (MRI) demonstrated that the lesion was hypointense on T1-weighted imaging (T1WI; with mixed signals of exudate in the mastoid cavity), hyperintense on T2-weighted imaging (T2WI), hypointense on T2 fluid-attenuated inversion recovery imaging (T2 FLAIR), and hypointense on diffusion-weighted imaging (DWI). Partial lesions were even contrast-enhanced (Figure 1).

The transmastoid approach was adopted for the exploration. A cyst-like lesion was observed in the tympanum along the course of the facial nerve. The fallopian canal at the geniculate ganglion and proximal tympanic segment of the facial nerve were eroded. Upon piercing the cyst, a massive amount of clear fluid leaked. Further inspection revealed that the fundus of the internal auditory meatus was defective, and the releasing fluid was CSF. The facial nerve trunk was separated into multiple filaments and laid freely in the CSF (Figure 1). After removing the cystic wall, a normal facial nerve stump was identified within the internal auditory meatus. CSF pressure rendered it difficult to simultaneously perform autologous nerve interposition grafting and repair of CSF otorrhea merely via the transmastoid approach; thus, a combined middle fossa approach surgery for nerve grafting was recommended to the boy’s parents; however, they declined because they were worried about the trauma and potential complications of craniotomy, such as infection, and they chose secondary facial-hypoglossal nerve anastomosis because its best outcome was the same as concurrent autologous nerve interposition grafting, which was HB grade III. Therefore, only CSF leakage was repaired, by packing the internal auditory meatus with the temporalis fascia, muscle, and fibrin glue (Supplementary Video 1).

Approximately 4 months postoperatively, the facial nerve function remained at HB grade VI, and facial-hypoglossal nerve anastomosis was performed for facial reanimation. A 2-year follow-up revealed no CSF otorrhea or meningitis, and facial function improved to HB grade IV. We assumed that the boy’s failure to improve to HB grade III was mainly because facial muscles usually began to undergo irreversible atrophy 12 months after denervation. His parents understood the pathophysiology of facial nerve rehabilitation; hence, they generally accepted the final result.

### 2.2. Case two

**Figure 2:**
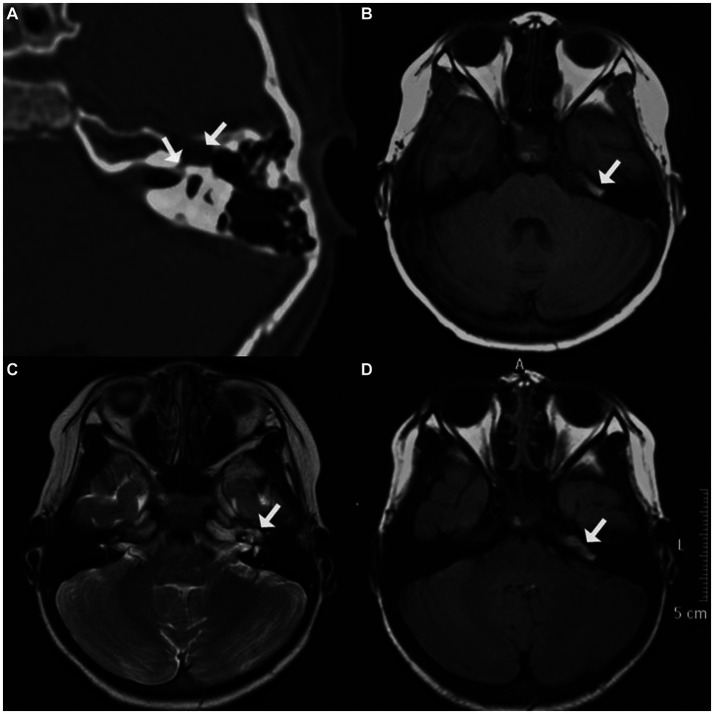
Imaging of case two. **(A)** Axial computed tomographic (CT) image displaying enlarged fallopian canal at the labyrinthine segment, a cystic lesion at geniculate ganglion, and defective adjacent middle cranial fossa floor; **(B)** mixed hypointensity and hyperintensity at the tympanic segment of the facial nerve on T1-weighted imaging (T1WI); **(C)** hyperintensity of the perigeniculate lesion on T2-weighted imaging (T2WI); **(D)** mixed hypointensity and isointensity at the tympanic segment on T2 fluid-attenuated inversion recovery imaging (T2 FLAIR).

A 5-year-old girl came to our center with left-sided HB grade V facial nerve paralysis. Before 6 months, she had a high fever, headache, and vomiting, and her left-sided facial nerve was paralyzed 2 days later. She was diagnosed with viral meningitis. After treatment with immunoglobulin, mannitol, and corticosteroids for approximately 3 weeks, she recovered from meningitis and intracranial hypertension symptoms; however, facial nerve paralysis did not improve even after 5 months of facial exercises; hence, she was transferred to our center. Pure-tone audiometry revealed that she also had moderate left-sided sensorineural hearing loss. The auditory brainstem response (ABR) revealed the disappearance of II–V waves, indicating that the distal segment of the acoustic nerve was injured. Further CT revealed an asymmetrically enlarged fallopian canal at the labyrinthine segment, a cystic lesion at the geniculate ganglion and proximal tympanic segment of the facial nerve, and a defective middle cranial fossa floor. MRI revealed mixed hypointensity and hyperintensity on T1WI, hyperintensity on T2WI, mixed hypointensity and isointensity on T2 FLAIR imaging, and hypointensity on DWI (Figure 2).

Exploration was conducted using the transmastoid approach. A cystic bulge was identified near the geniculate ganglion and proximal tympanic segment of the facial nerve. The floor of the middle cranial fossa was eroded; however, the dura mater remained intact. Upon removal of a small portion of the lesion, CSF emanated from the subarachnoid space through the internal auditory meatus. The compressed facial nerve atrophied and thinned but remained continuous. Dehiscence of the fundus of the internal auditory meatus and the floor of the middle cranial fossa was packed with temporalis fascia, muscle, and fibrin glue. After repairing the CSF otorrhea and decompressing the facial nerve, the facial nerve was covered with the temporalis fascia and a gelatin sponge.

No postoperative recurrence of CSF otorrhea and meningitis was observed during the 4-year follow-up. Facial function improved to HB grade IV; however, the left-sided sensorineural hearing loss could not be alleviated. The rehabilitation of chronic facial nerve paralysis and sensorineural hearing loss was difficult although dissatisfied, her parents generally accepted the outcomes.

## 3. Discussion

In a previous study of Gacek’s anatomy of 163 temporal bones, arachnoid cysts were normally limited to the internal auditory meatus by the fundus and fibrous ring; however, 12% still extended beyond the internal auditory meatus (1). Arachnoid cysts in the fallopian canal are rarely observed because they seldom cause clinical symptoms or are clearly imaged. Since the first case was reported in 1967, fewer than 20 patients have been reported to be symptomatic worldwide, of whom nearly all presented with CSF otorrhea (2). Cases presenting with facial nerve paralysis but without CSF otorrhea are extremely rare, and their clinical features are occasionally uncharacteristic when distinguishing them from facial nerve schwannomas.

The two patients included in this report experienced acute facial nerve paralysis after meningitis. Once an arachnoid cyst went through the fundus of the internal auditory canal, it could extend into the substance of the geniculate ganglion and dissect the nerve fascicles or laterally compress the nerve trunk (1, 2), which were the pathology of the two cases, respectively. However, facial function could be maintained normally with as low as 10% of nerve fibers; therefore, elevated CSF pressure secondary to meningitis may be the last straw that leads to paralysis (6). In the world literature, only three patients with fallopian canal arachnoid cysts reportedly develop facial nerve paralysis; the facial nerve paralysis of one patient finally developed to HB grade III after several recurrent CSF otorrhea and meningitis episodes. One patient had HB grade II facial paralysis after idiopathic intracranial hypertension, and the other had transient facial paralysis before meningitis (3–5) (Table 1).

**Table 1:** Reported fallopian canal arachnoid cyst with facial nerve paralysis.

Author	Year	Age	Sex	Laterality	Site	Principal disease	Facial palsy	Hearing loss	Treatment
Isaacson et al. (3)	2002	37	Male	Left	Perigeniculate ganglion	Recurrent cerebrospinal fluid otorrhea and meningitis	HB grade III	Mixed	Surgery
Brackmann et al. (4)	2007	8	Male	Right	Geniculate ganglion	Idiopathic intracranial hypertension	HB grade II	Sensorineural	Medicine
Sagardoy et al. (5)	2017	NS	NS	NS	Geniculate ganglion	Meningitis	Transient	Normal	Surgery

NS, not specified; HB, House–Brackmann.

Imaging in these two cases resembled a schwannoma, which posed a challenge to the diagnosis of a fallopian canal arachnoid cyst. For an arachnoid cyst, the principal content is CSF; therefore, it should exhibit the same signals as CSF on MR, with hypointensity on T1WI and T2 FLAIR, hyperintensity on T2WI, no diffusion restriction, and no contrast enhancement. However, in case one, part of the lesion was uncharacteristically contrast-enhanced, which should have been the main differential characteristic from a schwannoma; in case two, there was misleading mixed hyperintensity on T1WI and mixed isointensity on T2 FLAIR, which resembled signals of schwannoma (7). The reason may be that, although an arachnoid cyst is a duplication or splitting of the arachnoid layer, it can occasionally be loculated by septations; the content is similar but not equal to that of the CSF (8), and contrast enhancement occasionally appears on the cyst wall or adjacent tissues when infected. Since there is no obvious difference in clinical symptoms and signs between a schwannoma and a fallopian canal arachnoid cyst, despite some variations, differentiation mainly relies on enhanced MRI because the signals of the main body of arachnoid cysts are similar to those of the CSF.

The treatment of arachnoid cysts with facial nerve paralysis without CSF otorrhea can be challenging. Once the arachnoid cyst is removed, CSF otorrhea is confirmed, and repair of CSF otorrhea occurs before facial nerve management. Repair of CSF otorrhea may be complicated; even five repeated surgeries could not reportedly help thoroughly control the recurrence of CSF otorrhea in a patient (3). Packing the dehiscence of CSF otorrhea could potentially exacerbate facial nerve paralysis (9, 10). Therefore, infections or factors contributing to elevated CSF pressure should first be addressed (11). Exploration can be performed if the facial nerve paralysis is severe or complete. In cases of mild or moderate paralysis, exploration should be measured and discussed with the patient (12). In addition, an appropriate surgical approach is also an important issue to concern, especially when facial nerve repair is needed. For case one, transmastoid combined with the middle fossa approach may be a better choice because it could potentially facilitate concurrent autologous interposition grafting.

These two cases did not receive an accurate diagnosis of facial nerve paralysis until they came to our center because meningitis confused the diagnosis of a fallopian canal arachnoid cyst, and delayed diagnosis and surgery potentially decreased the probability of a satisfactory recovery of facial function (13, 14). In addition, differentiating arachnoid cysts from schwannomas and sufficient preparation is vital before surgery because repair of CSF otorrhea and facial nerve concurrently could occasionally be challenging.

## 4. Conclusion

Arachnoid cysts in the fallopian canal are exceedingly rare causes of facial nerve paralysis and occasionally resemble schwannomas. Differentiation mainly relies on enhanced MRI, and the signals of arachnoid cysts are similar to those of the CSF despite some variations. For treatment, factors contributing to elevated CSF pressure should first be addressed; exploration is suitable for cases with severe or complete paralysis, and for those with mild or moderate paralysis, exploration should be measured because repair of CSF otorrhea during surgery can occasionally be complex, and facial nerve paralysis may even be exacerbated after packing in the dehiscence of CSF otorrhea.

## Data availability statement

The original contributions presented in the study are included in the article/Supplementary material, further inquiries can be directed to the corresponding author.

## Ethics statement

The studies involving human participants were reviewed and approved by the Ethics Committee of Chinese PLA General Hospital. Written informed consent to participate in this study was provided by the participants' legal guardian/next of kin. Written informed consent was obtained from the minor(s)' legal guardian/next of kin for the publication of any potentially identifiable images or data included in this article.

## Author contributions

JS: acquisition of materials and manuscript drafting. WH: direct patient management and data interpretation. All authors contributed to the article and approved the submitted version.

## Funding

This study was supported by grants from the Open Program of the National Clinical Research Center for Otorhinolaryngologic Diseases (202200007), the National Natural Science Foundation of China (NSFC#81770992), and the Innovation Cultivation Fund of the Sixth Medical Center of the Chinese PLA General Hospital (CXPY202113).

## Conflict of interest

The authors declare that the research was conducted in the absence of any commercial or financial relationships that could be construed as a potential conflict of interest.

## Publisher’s note

All claims expressed in this article are solely those of the authors and do not necessarily represent those of their affiliated organizations, or those of the publisher, the editors and the reviewers. Any product that may be evaluated in this article, or claim that may be made by its manufacturer, is not guaranteed or endorsed by the publisher.
